# Analysis of Maxwell nanofluid model with convective variations, variable-density and heat generation in a porous medium

**DOI:** 10.1186/s11671-026-04947-2

**Published:** 2026-09-29

**Authors:** Mohammed Alrehili

**Affiliations:** Department of Mechanical Engineering, Faculty of Engineering, University of Tabuk, Tabuk, 71491 Saudi Arabia

**Keywords:** Maxwell nanofluid, Variable density, Convective boundary condition, Ohmic heating, Heat generation, Porous medium

## Abstract

This study introduces a novel analysis of Maxwell nanofluid flow over a stretching surface, integrating multiple coupled physical mechanisms previously overlooked in the literature. The comprehensive model incorporates boundary convection, internal heat generation, Ohmic heating, temperature-dependent transport properties (viscosity, density, and diffusivity), and viscous dissipation effects. This holistic approach distinguishes the current work from earlier studies, which have not simultaneously examined the coupled effects of these interacting mechanisms within a unified Maxwell nanofluid model. A mathematical model is developed and solved numerically using similarity transformations, the shooting method, and the fourth-order Runge-Kutta scheme. Validation against established benchmarks confirms the method’s reliability. Further, the results demonstrate the coupled influence of key parameters on flow, thermal, and concentration profiles, along with their effects on skin friction, heat transfer (Nusselt number), and mass transfer (Sherwood number) rates. This study addresses a significant research void while providing useful theoretical guidance for the design and optimization of polymer processing, heat exchanger systems, and nanofluid-based thermal management technologies. A central revelation of this research is the dual role of temperature-sensitive density: it simultaneously amplifies thermal distribution while attenuating both nanoparticle concentration and fluid velocity. This parameter further enhances thermal and mass transfer rates while increasing the surface drag, indicating its important role in the coupled transport behavior of Maxwell nanofluids. Additionally, heat generation and convective mechanisms, as anticipated, elevate temperature profiles but inhibit flow momentum, resulting in suppressed velocity and refined control over transport behavior. Quantitatively, increasing the porous parameter enhances the skin-friction coefficient, local Nusselt number, and local Sherwood number by approximately 20.8%, 45.6%, and 3.0%, respectively, while increasing the density parameter raises these quantities by about 75.1%, 79.8%, and 69.2%, respectively. These quantitative improvements highlight the theoretical potential of the proposed model to support the design and optimization of heat exchangers, polymer processing, and nanofluid-based thermal management systems, while accounting for the associated increase in surface drag.

## Introduction

The analysis of non-Newtonian fluid dynamics has attracted considerable interest owing to its essential applications in industrial and technological operations-including polymer processing, surface coating, and thermal management systems. This practical relevance has stimulated substantial research aimed at elucidating the underlying transport phenomena of these fluids under diverse physical conditions. As a widely recognized non-Newtonian fluid model, the Maxwell formulation is particularly valued for its capacity to represent elastic behavior and stress relaxation phenomena in complex flow environments. Contemporary studies have markedly advanced the theoretical and practical understanding of this category of non-Newtonian fluids. Recent research in non-Newtonian Maxwell fluid dynamics has expanded significantly across multiple interdisciplinary fronts. Abdelsalam et al. [1] conducted a comparative assessment of rheological properties over permeable stretching surfaces, emphasizing interactions between fluid microstructure and boundary conditions. Parallelly, Venkatesh et al. [2] investigated augmented thermal and species transport by incorporating nanoparticles, radiative heat transfer, and chemical reaction effects. Mandal et al. [3] examined mixed convection in magnetohydrodynamic flow under time-dependent stretching, whereas Gurejala et al. [4] analyzed the influence of viscous dissipation in electromagnetic nanofluid systems. Further extending the scope, Basit et al. [5] introduced a model coupling bioconvection with Darcy-Forchheimer porous resistance and Lorentz forces, offering new perspectives on flow-porous media interactions. In a contemporary contribution, Zeeshan et al. [6] explored hydrodynamic stability in thermally magnetized flow over unsteady stretching geometries, enriching the theoretical framework for time-dependent fluid phenomena. Together, these studies reflect the expanding scope of current investigations and confirm the continued relevance of non-Newtonian transport phenomena in advancing both fundamental theory and technological applications.

The importance of nanofluids has grown significantly owing to their superior thermal conductivity, which makes them ideal for application in heat transfer, thermal management, energy systems, cooling systems, and processing technologies. It is, therefore, not surprising that studies on the flow, heat, and mass transfer properties of nanofluids have been carried out extensively due to their versatility in various fluid models and configurations. Anwar [7] used fractional derivatives and fractional relaxation times in the Buongiorno model of heat and mass transfer in viscoelastic nanofluids. The study by Irfan et al. [8] was based on the mass flux principle for the estimation of convective heat transfer mechanism in Carreau nanofluid taking into account nonlinear mixed convection effects. On the other hand, Sarfraz and Khan [9] conducted the analysis of second-law analysis of hybrid nanofluid with silver and titania particles having heat generation and buoyancy forces over an inclined stretching sheet. Moreover, Ohmic heating and activation energy effects were also taken into account in the study of Jeffrey nanofluid flow with gyrotactic microorganisms by Muhammad et al. [10]. In Rajhi et al. [11], the thermal study of tetra-nanofluid is done within a vertically aligned needle with both convection and porous media effects. In addition to that, Gizachew et al. [12] performed a study on time-dependent MHD Williamson nanofluid flow with an inclined stretching surface within a porous medium. The study on the bioconvective Darcy-Forchheimer flow of Ree-Eyring nanofluid flow with velocity and thermal slip effects was performed by Puneeth et al. [13]. Furthermore, the triple-diffusive radiated Prandtl nanofluid flow with multiple thermal effects and varying thermophysical properties was modeled mathematically by Khan et al. [14]. Oldroyd-B nanofluid flow in a non-inertial frame of reference was studied using Cattaneo-Christov heat-flux model by Yasir et al. [15]. Finally, Majeed et al. [16] recently did the study on bioconvective hydromagnetic Ree-Eyring nanofluid flow with Cattaneo-Christov heat-flux and gyrotactic microorganisms with Darcy-Forchheimer resistance.

Boundary interactions play a critical role in fluid transport phenomena, as they exert direct control over heat and mass transfer efficiencies in numerous engineering and industrial applications. This fundamental importance has prompted increased investigative focus on fluid dynamic behavior under realistic physical conditions, including non-ideal thermal boundary conditions and slip flow effects, to more accurately represent practical operational environments. Illustrating this trend, Baag et al. [17] examined the effects of surface stretching and internal heat generation on nanofluid dynamics, yielding valuable insights into thermal performance optimization. Building on the study of non-Newtonian behavior, Abbas et al. [18] examined Powell-Eyring fluid flow over a stretching sheet, integrating velocity slip and viscous dissipation to elucidate the intricate dynamics of viscoelastic fluids. Further expanding this research direction, Dhlamini et al. [19] analyzed second-grade nanofluids with bioconvective effects, highlighting the synergistic interaction between microbial motility and hydrodynamic boundaries. In a related investigation, Gundagani et al. [20] explored nano-enhanced Powell-Eyring fluid transport within porous media, focusing on the coupling of magnetohydrodynamic forces and thermal energy transfer mechanisms. Francis et al. [21] expanded the computational modeling of bioconvective nanofluids over conical surfaces, revealing how magnetic fields influence hydrodynamic stability. In the most recent contribution, Panda et al. [22] investigated hybrid nanofluids exhibiting tangent hyperbolic rheological behavior, incorporating simultaneous effects of magnetism, porous media resistance, and wall slip conditions. Together, these studies underscore the critical importance of boundary-mediated transport phenomena and illustrate the growing sophistication of contemporary models in pushing the frontiers of thermal and mass transfer science.

Fluid density plays a pivotal role in heat and mass transfer phenomena, significantly influencing thermal stability, buoyancy-induced convection, and overall energy and species transport efficiency in nanofluid applications. Recognizing this importance, researchers have increasingly explored its behavior under varied physical constraints. For instance, Yadav et al. [23] analyzed the direct dependence of nanofluid density on temperature and concentration variations, providing a foundational understanding for subsequent research. Expanding on this, Abbas et al. [24] integrated density variation and thermal radiation into a magnetohydrodynamic Sakiadis flow model, demonstrating their synergistic effect on transport dynamics. Gamaoun et al. [25] expanded upon this research direction by evaluating heat transfer in nanofluids along stretching surfaces under magnetic exposure, utilizing the Keller Box numerical technique to achieve high computational accuracy. Concurrently, Ullah et al. [26] explored the behavior of chemically reactive nanofluids, demonstrating the coupled influence of density gradients and viscous dissipation on the evolution of thermal and concentration boundary layers. Further contributions include Ullah et al. [27], who studied Carreau nanofluid flows with chemical reactions and heat generation under realistic heating conditions, and Khedher et al. [28], who emphasized conductivity and density effects on second-grade nanofluids over polymer surfaces. These studies underscore the fundamental influence of density-dependent phenomena on nanofluidic transport behavior, driving ongoing research to optimize thermohydraulic models for cutting-edge engineering systems.

Although previous studies have significantly advanced the understanding of Maxwell nanofluid transport under various physical conditions, they have primarily examined thermal convective boundary conditions, internal heat generation, viscous dissipation, Joule heating, and temperature-dependent thermophysical properties separately or in limited combinations. Consequently, the coupled influence of these interacting mechanisms on flow, heat, and mass transfer remains insufficiently understood. To address this gap, the present study develops a unified mathematical model that simultaneously incorporates these interacting mechanisms. A comprehensive review of the available literature further indicates that no previous study has simultaneously incorporated all of these interacting mechanisms within a single Maxwell nanofluid framework, thereby confirming the originality and necessity of the present investigation. The novelty of the present work lies in providing a comprehensive analysis of their coupled effects within a single Maxwell nanofluid framework, thereby offering a deeper understanding of the underlying transport characteristics and supplying useful guidance for the design and optimization of advanced thermal engineering systems.

## Flow analysis

**Fig. 1:**
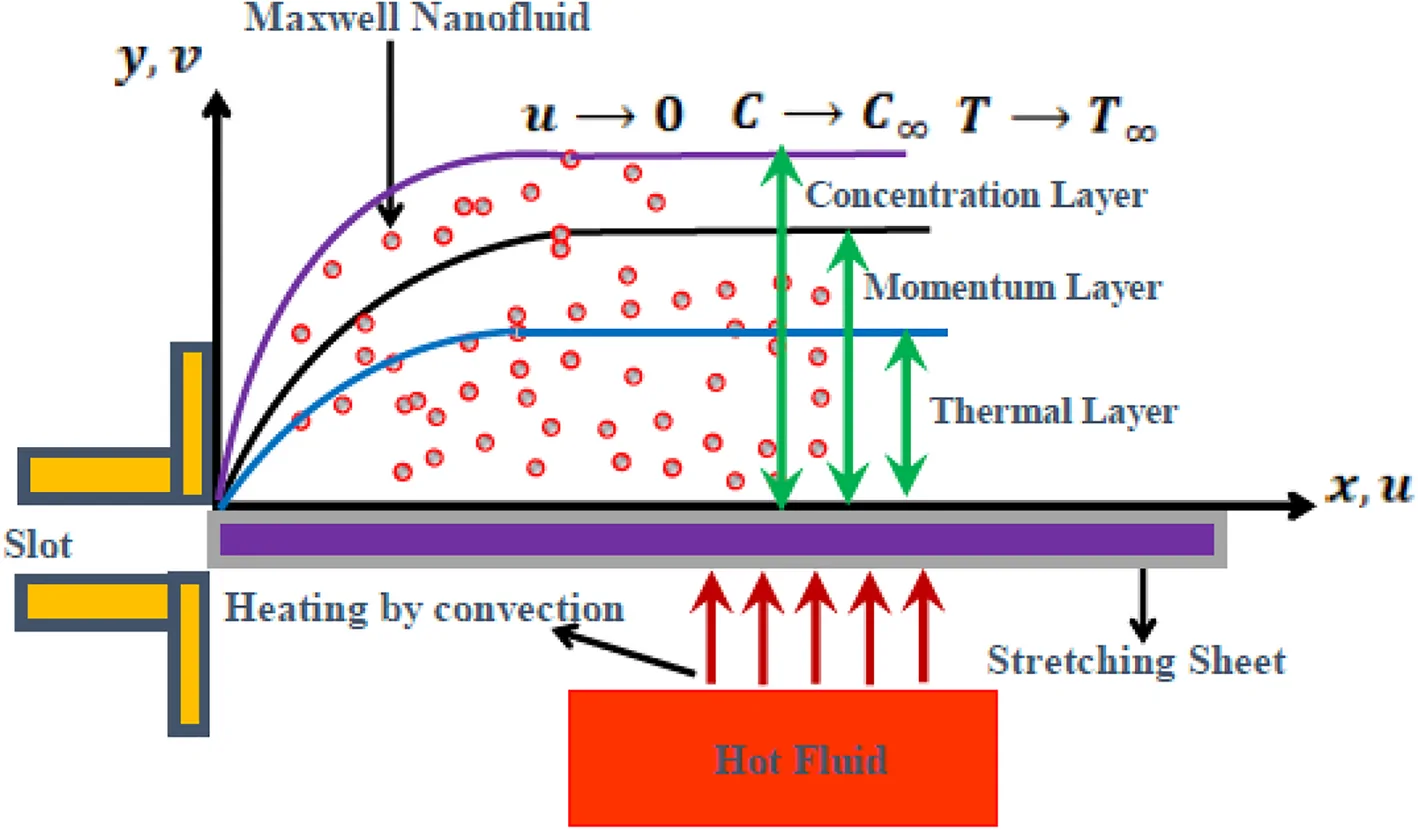
The coordinate flow geometry system

This section outlines the mathematical framework governing the flow, heat, and mass transfer of a non-Newtonian Maxwell fluid across a stretching sheet. The model (Fig. 1) incorporates temperature-dependent density and viscosity, along with thermophysical properties such as thermal conductivity and mass diffusivity that vary with both temperature and solute concentration. The stretching-sheet configuration considered in the present study is widely adopted to model boundary-layer transport phenomena encountered in polymer extrusion, continuous coating, wire drawing, and cooling processes. Although the stretching-sheet geometry is well established, the novelty of the present study lies in employing this configuration as a unified platform to simultaneously investigate the coupled effects of thermal convective boundary conditions, internal heat generation, Ohmic heating, viscous dissipation, and temperature-dependent transport properties in Maxwell nanofluids. This distinguishes the present model from previous studies that considered these mechanisms individually or in limited combinations. Consequently, the proposed configuration provides new physical insights into the coupled momentum, heat, and mass transfer characteristics under realistic operating conditions. Therefore, the proposed model represents a realistic engineering configuration for advanced thermal processing and nanofluid cooling systems, where these physical mechanisms may coexist simultaneously.

The momentum equation accounts for the impact of viscous, magnetic, porous, and the couple stress which based on the Maxwell type, whereas the energy equation includes convection, viscous dissipation, heat generation phenomenon, Ohmic heating, and nanoparticle contributions such as Brownian motion and thermophoresis. The concentration equation further reflects the impact of variable diffusion processes. Appropriate boundary conditions are imposed at the sheet surface with convection impact and at infinity to complete the problem formulation. For density-varying $\bar{\rho }$ incompressible flows in planar geometry, the following Eq. (1) maintains mass continuity through velocity components *u* (*x* − direction) and *v* (*y* − direction) [28]:

1 $$\dfrac{\partial }{\partial y}\left(\bar{\rho }\,v\right)=-\dfrac{\partial }{\partial x}\left(\bar{\rho }\,u\right).$$

Further, the system incorporates density-temperature $\bar{\rho }=\frac{\rho }{{\rho }_{c}}$ coupling through a normalized exponential formulation in the following equation (2), with Γ_1_ quantifying the thermal sensitivity parameter, thus introducing thermophysical property variations into the governing equations, as described below. Furthermore, the analysis employs Ψ(*x*, *y*) as the fundamental variable, whose definitional properties intrinsically maintain fluid continuity [28]:

2 $$\bar{\rho }=\dfrac{\rho }{{\rho }_{c}}={e}^{-{{\rm{\Gamma }}}_{1}\left(\dfrac{{T}_{\infty }-T}{{T}_{\infty }-{T}_{f}}\right)}=-\dfrac{1}{v}\,\left(\dfrac{\partial {\rm{\Psi }}(x,y)}{\partial x}\right)=\dfrac{1}{u}\,\left(\dfrac{\partial {\rm{\Psi }}(x,y)}{\partial y}\right),$$

where *ρ*_*c*_ is the constant density, *T* is the temperature, *T*_*∞*_ is the constant ambient temperature and *T*_*f*_ is the fluid temperature of heating fluid. Also, our formulation presents the momentum equation for 2D flow of a non-Newtonian Maxwell model in porous spaces that controlled by the coefficient of permeability *k*_0_ and under transverse magnetic-field influence with intensity *B*_0_, while considering temperature-dependent viscous properties [29]:

3 $$\begin{array}{lll}\rho \left[u\,\dfrac{\partial u}{\partial x}+v\,\dfrac{\partial u}{\partial y}\right]+\sigma \,{B}_{0}^{2}\,u+u\left(\dfrac{\mu }{{k}_{0}}\right)&=&\dfrac{\partial \mu }{\partial y}\,\dfrac{\partial u}{\partial y}+\mu \,\left(\dfrac{{\partial }^{2}u}{\partial {y}^{2}}\right)\\&& -\rho \,{\beta }_{0}\left\{{v}^{2}\dfrac{{\partial }^{2}u}{\partial {y}^{2}}+2uv\dfrac{{\partial }^{2}u}{\partial x\partial y}+{u}^{2}\dfrac{{\partial }^{2}u}{\partial {x}^{2}}\right\},\end{array}$$

where *σ* is the electrical conductivity, *β*_0_ is the relaxation parameter and *μ* is the fluid viscosity, which assumed to vary with temperature according to the following equation [29, 30]:

4 $$\mu ={\mu }_{0}\,{e}^{-{{\rm{\Gamma }}}_{2}\left(\dfrac{{T}_{\infty }-T}{{T}_{\infty }-{T}_{f}}\right)},$$

where Γ_2_ is the parameter of viscosity and *μ*_0_ is the reference constant viscosity. Likewise, the following energy equation (5) governs the heat transfer mechanism in a Maxwell fluid flow through a porous medium with variable thermal conductivity *κ*(*T*) under magnetic heating which introduced through the Joule dissipation term $\sigma \,{B}_{0}^{2}\,{u}^{2}$, the viscous dissipation phenomenon, the volumetric heat generation that represented mathematically by ${Q}^{{\prime\prime} {\prime} }$, both thermophoresis and Brownian motion properties which are incorporated via the Buongiorno model parameters *D*_*T*_ and *D*_*B*_[29]:

5 $$\begin{array}{lll}\rho {c}_{p}\left(u\dfrac{\partial T}{\partial x}+v\dfrac{\partial T}{\partial y}\right)+\rho \,{\beta }_{0}\left(2uv\dfrac{\partial u}{\partial x}\dfrac{\partial u}{\partial y}+{v}^{2}{\left(\dfrac{\partial u}{\partial y}\right)}^{2}\right)&=&\sigma \,{B}_{0}^{2}\,{u}^{2}+\dfrac{\partial \kappa }{\partial y}\,\dfrac{\partial T}{\partial y}+\kappa \,\dfrac{{\partial }^{2}T}{\partial {y}^{2}}+{Q}^{{\prime\prime} {\prime} }\\ && +\rho \,{c}_{p}\,\tau \left\{\dfrac{{D}_{T}}{{T}_{\infty }}\left(\dfrac{\partial T}{\partial y}\right)+{D}_{B}\dfrac{\partial C}{\partial y}\right\}\dfrac{\partial T}{\partial y}+\mu {\left(\dfrac{\partial u}{\partial y}\right)}^{2},\end{array}$$

where *τ* is the ratio of nanoparticle heat capacity to Maxwell base fluid heat capacity. Here, the thermal conductivity *κ* is assumed to satisfy the following equation [29]:

6 $$\kappa ={\kappa }_{0}\,\left[1+{{\rm{\Gamma }}}_{3}\,\left(\dfrac{{T}_{\infty }-T}{{T}_{\infty }-{T}_{f}}\right)\right],$$

where Γ_3_ is the factor of conductivity and *κ*_0_ is the constant conductivity value. Additionally, the nanoparticle mass transfer mechanism is characterized below by a convection-diffusion balance in the concentration field equation as [30]:

7 $$\begin{array}{l}u\dfrac{\partial C}{\partial x}+v\dfrac{\partial C}{\partial y}=\dfrac{\partial }{\partial y}\,\left({D}_{B}\,\dfrac{\partial C}{\partial y}\right)+\dfrac{{D}_{T}}{{T}_{\infty }}\dfrac{{\partial }^{2}T}{\partial {y}^{2}}.\end{array}$$

In the present study, we further assume that the diffusion coefficient *D*_*B*_ is a linear function of concentration as described below [31]:

8 $${D}_{B}={D}_{0}\left[1+{{\rm{\Gamma }}}_{4}\,\left(\dfrac{{C}_{\infty }-C}{{C}_{\infty }-{C}_{w}}\right)\right],$$

where Γ_4_ is the diffusion parameter, *C*_*∞*_ is the constant concentration away the sheet and *C*_*w*_ is the nanoparticle concentration beside the sheet. The temperature-dependent models for density (2), viscosity (4), conductivity (6), and diffusivity (8) are adopted as physically reasonable approximations for nanofluids subjected to moderate temperature variations. Within the considered parameter ranges, these properties remain finite and physically meaningful, allowing the model to capture temperature-induced variations in transport behavior without introducing unrealistic property changes. These assumptions are consistent with commonly adopted temperature-dependent property models reported in previous nanofluid studies. Our governing equations require completion through the following auxiliary conditions that taking into account the thermal condition which follows a convective boundary formulation, where the heat flux mechanism $\kappa \,\left(\frac{\partial T}{\partial y}\right)$ is proportional to the temperature difference with proportionality constant ϒ_*f*_. Here we observe the following conditions [29]:

9 $$u={u}_{w},\quad C={C}_{w},\quad \kappa \,\left(\dfrac{\partial T}{\partial y}\right)=-{{\rm{\Upsilon }}}_{f}\left(T-{T}_{f}\right),\quad v=-{v}_{w}\quad at\quad y=0,$$

10 $$u\to 0,\quad C\to {C}_{\infty },\quad T\to {T}_{\infty },\quad as\quad y\to \infty ,$$

here, we must observe that the boundary conditions represent a stretching surface moving with velocity *u*_*w*_, subjected to wall suction/blowing *v*_*w*_, a prescribed wall nanoparticle concentration *C*_*w*_, and a convective heating condition characterized by the heat transfer coefficient ϒ_*f*_. Far from the surface, the fluid is assumed to be quiescent and attains the ambient temperature *T*_*∞*_ and nanoparticle concentration *C*_*∞*_. Now and through the following similarity quantities, we can establish the dimensionless framework, concentration ϕ(*η*), dimensionless stream function *f*(*η*), and temperature *θ*(*η*) as follows [32]:

11 $$\eta =\sqrt{\dfrac{a}{\nu }}\int_{0}^{y}\bar{\rho }\,dy,\quad f(\eta )=\dfrac{{\rm{\Psi }}(x,y)}{x\,\sqrt{a\,\nu }},\quad \phi (\eta )=\left(\dfrac{{C}_{\infty }-C}{{C}_{\infty }-{C}_{w}}\right),\quad \theta (\eta )=\left(\dfrac{{T}_{\infty }-T}{{T}_{\infty }-{T}_{f}}\right).$$

We now turn to the analysis of non-uniform heat generation, represented mathematically by the symbol ${Q}^{{\prime\prime} {\prime} }$. The governing expression for this mechanism is derived below, following the approach established in [33]:

12 $${Q}^{{\prime\prime} {\prime} }=\dfrac{{\gamma }_{0}}{{e}^{\eta }},$$

where *γ*_0_ corresponds to the steady-state heat generation intensity. Employing the dimensionless quantities defined in Eq. (11), the governing system of equations-from (1) through (7)-is transformed into the following non-dimensionalized form:

13 $$\begin{array}{ll}&{e}^{-({{\rm{\Gamma }}}_{1}+{{\rm{\Gamma }}}_{2})\theta }\,\left\{{f}^{{\prime\prime} {\prime} }-\left({{\rm{\Gamma }}}_{1}+{{\rm{\Gamma }}}_{2}\right)\,{\theta }^{{\prime} }\,{f}^{{\prime\prime} }\right\}+\beta \,\left\{\left(2\,{f}^{{\prime} }+{{\rm{\Gamma }}}_{1}\,f\,{\theta }^{{\prime} }\right){f}^{{\prime\prime} }-f\,{f}^{{\prime\prime} {\prime} }\right\}\,f\\ & -{f}^{{\prime} 2}+f\,{f}^{{\prime\prime} }-M\,{e}^{{{\rm{\Gamma }}}_{1}\theta }\,{f}^{{\prime} }-\delta \,{e}^{({{\rm{\Gamma }}}_{1}-{{\rm{\Gamma }}}_{2})\theta }\,{f}^{{\prime} }=0,\end{array}$$

14 $$\begin{array}{ll}&\dfrac{{e}^{-{{\rm{\Gamma }}}_{1}\theta }}{\Pr }\,\left\{-\left({{\rm{\Gamma }}}_{1}-{{\rm{\Gamma }}}_{3}+{{\rm{\Gamma }}}_{1}\,{{\rm{\Gamma }}}_{3}\,\theta \right)\,{\theta }^{{\prime} 2}+\left(1+{{\rm{\Gamma }}}_{3}\,\theta \right)\,{\theta }^{{\prime\prime} }\right\}+f\,{\theta }^{{\prime} }+M\,Ec\,{e}^{{{\rm{\Gamma }}}_{1}\theta }\,{f}^{{\prime} 2}+\gamma \,{e}^{{{\rm{\Gamma }}}_{1}\theta -\eta }\\ &+Ec\,{f}^{{\prime\prime} }\,\left\{{e}^{-({{\rm{\Gamma }}}_{1}+{{\rm{\Gamma }}}_{2})\theta }\,{f}^{{\prime\prime} }+\beta \,f\,\left(2\,{f}^{{\prime} 2}-f\,{f}^{{\prime\prime} }\right)\right\}+{e}^{-2{{\rm{\Gamma }}}_{1}\theta }\,\left({\xi }_{t}\,{\theta }^{{\prime} 2}+{\xi }_{r}\,{\phi }^{{\prime} }\,{\theta }^{{\prime} }\right)=0,\end{array}$$

15 $$\begin{array}{ll}&\dfrac{1}{Sc}\,{e}^{-2{{\rm{\Gamma }}}_{1}\theta }\,\left\{-{{\rm{\Gamma }}}_{1}\,\left(1+{{\rm{\Gamma }}}_{4}\,\phi \right)\,{\phi }^{{\prime} }\,{\theta }^{{\prime} }+{{\rm{\Gamma }}}_{4}\,{\phi }^{{\prime} 2}+\left(1+{{\rm{\Gamma }}}_{4}\,\phi \right)\,{\phi }^{{\prime\prime} }\right\}+f\,{\phi }^{{\prime} }\\ &+\left(\dfrac{{\xi }_{t}}{Sc\,{\xi }_{r}}\right)\,{e}^{-2{{\rm{\Gamma }}}_{1}\theta }\,\left({\theta }^{{\prime\prime} }-{{\rm{\Gamma }}}_{1}\,{\theta }^{{\prime} 2}\right)=0,\end{array}$$

where the following boundary conditions are enforced:

16 $${f}^{{\prime} }=1,\quad \phi =1,\quad {\theta }^{{\prime} }={\rm{\Upsilon }}\,{e}^{{{\rm{\Gamma }}}_{1}\theta }\,\left(\dfrac{1-\theta }{1+{{\rm{\Gamma }}}_{3}\,\theta }\right),\quad f=\omega \,{e}^{-{{\rm{\Gamma }}}_{1}\theta }\quad at\quad \eta =0,$$

17 $${f}^{{\prime} }\to 0,\quad \phi \to 0,\quad \theta \to 0\quad as\quad \eta \to \infty .$$

At this stage, we finalize the section by introducing the dimensionless governing parameters, defined as follows:

$$\left\{\begin{array}{ll}\beta ={\beta }_{0}\,a&\,\text{Maxwell parameter,}\,\\ \omega =\dfrac{{v}_{w}}{\sqrt{a\,\nu }}&\,\text{Suction parameter,}\,\\ {\rm{\Upsilon }}=\dfrac{{{\rm{\Upsilon }}}_{f}}{{\kappa }_{0}\,\sqrt{\dfrac{a}{\nu }}}&\,\text{Convection parameter,}\,\\ {\xi }_{r}=\dfrac{\tau {D}_{0}\left({C}_{w}-{C}_{\infty }\right)}{\nu }&\,\text{Brownian motion parameter,}\,\\ \gamma =\dfrac{{\gamma }_{0}}{a\,({T}_{f}-{T}_{\infty })\,{\rho }_{c}\,{c}_{p}}&\,\text{Heat generation parameter,}\,\\ \Pr =\dfrac{\mu {c}_{p}}{\kappa }&\,\text{Prandtl number,}\,\\ Sc=\dfrac{\nu }{{D}_{0}}&\,\text{Schmidt number,}\,\\ {\xi }_{t}=\dfrac{\tau {D}_{T}\left({T}_{f}-{T}_{\infty }\right)}{{T}_{\infty }\,\nu }&\,\text{Thermophoresis parameter,}\,\\ M=\dfrac{\sigma \,{B}_{0}^{2}}{{\rho }_{c}\,a}&\,\text{Magnetic field parameter,}\,\\ Ec=\dfrac{{u}_{w}}{{c}_{p}\left({T}_{w}-{T}_{\infty }\right)}&\,\text{Eckert number,}\,\\ \delta =\dfrac{\mu }{{\rho }_{c}\,a\,{k}_{0}}&\,\text{Porous parameter}\,\end{array}\right.$$

A comprehensive evaluation of the model necessitates examining the effects of its principal parameters on the rates of heat and mass transfer, expressed as $S{h}_{x}{\Re }_{x}^{\frac{-1}{2}}$ and $N{u}_{x}{\Re }_{x}^{\frac{-1}{2}}$ as well as on the skin friction coefficient, denoted as $C{f}_{x}{\Re }_{x}^{\frac{1}{2}}$. These dimensionless quantities are defined as follows:

18 $$C{f}_{x}R{e}_{x}^{\dfrac{1}{2}}=-\left[{f}^{{\prime\prime} }(0)-\beta \,{e}^{{{\rm{\Gamma }}}_{1}\theta (0)}\,\left(-2\,f(0)\,{f}^{{\prime} 2}(0)+{f}^{2}(0)\,{f}^{{\prime\prime} }(0)\right)\right],$$

19 $$S{h}_{x}{\Re }_{x}^{\dfrac{-1}{2}}=-\phi {\prime} (0),\quad N{u}_{x}{\Re }_{x}^{\dfrac{-1}{2}}=-{\theta }^{{\prime} }(0).$$

## Solution methodology

**Fig. 2:**
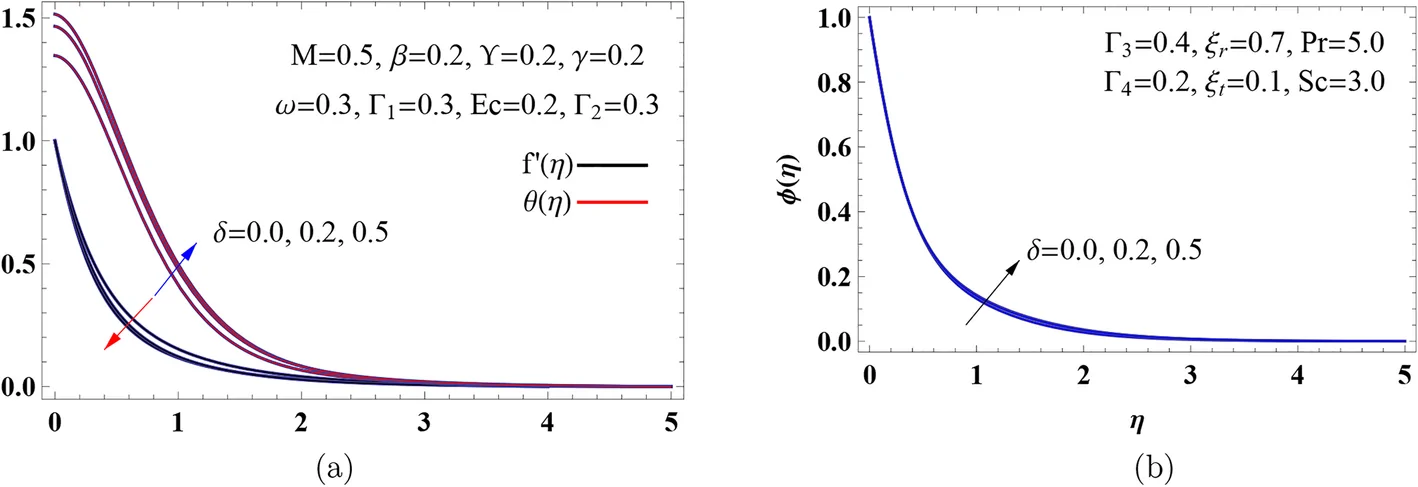
**a** Variation of *δ* on *θ*(*η*) and ${f}^{{\prime} }(\eta )$. **b** Variation of *δ* on ϕ(*η*)

**Fig. 3:**
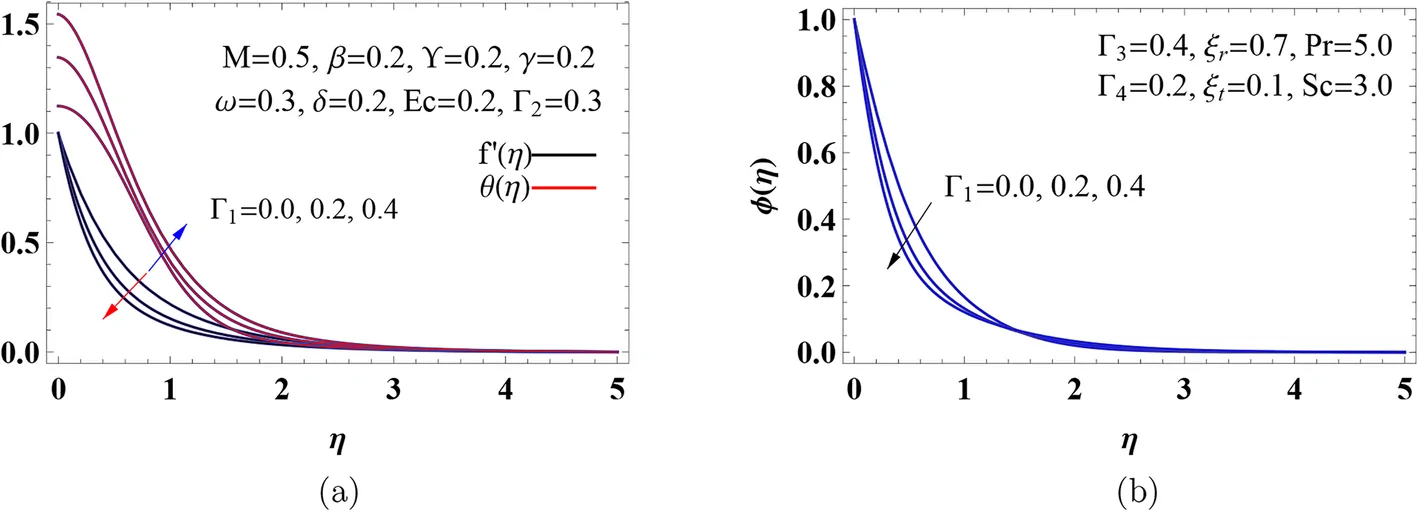
**a** Variation of Γ_1_ on *θ*(*η*) and ${f}^{{\prime} }(\eta )$. **b** Variation of Γ_1_ on ϕ(*η*)

**Fig. 4:**
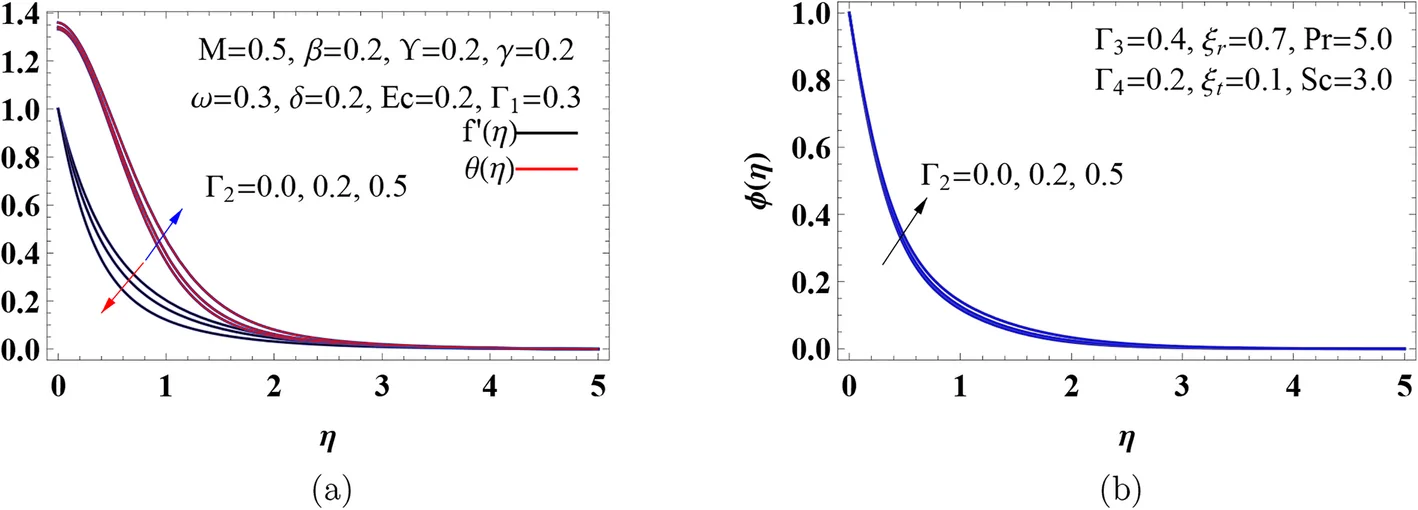
**a** Variation of Γ_2_ on *θ*(*η*) and ${f}^{{\prime} }(\eta )$. **b** Variation of Γ_2_ on ϕ(*η*)

**Fig. 5:**
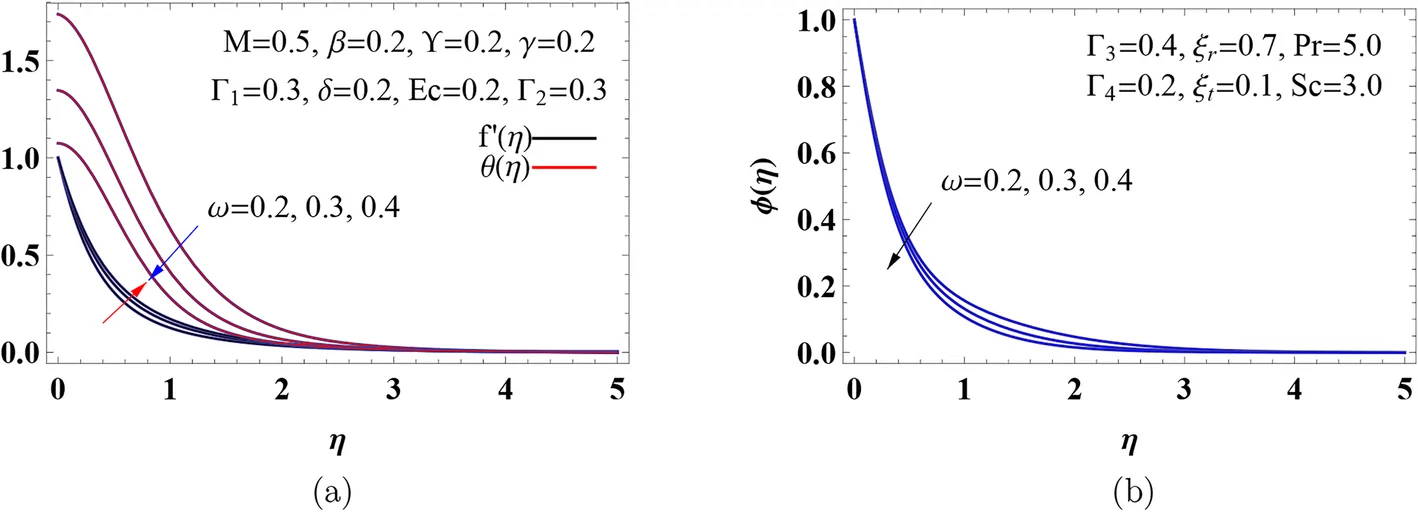
**a** Variation of *ω* on *θ*(*η*) and ${f}^{{\prime} }(\eta )$. **b** Variation of *ω* on ϕ(*η*)

**Fig. 6:**
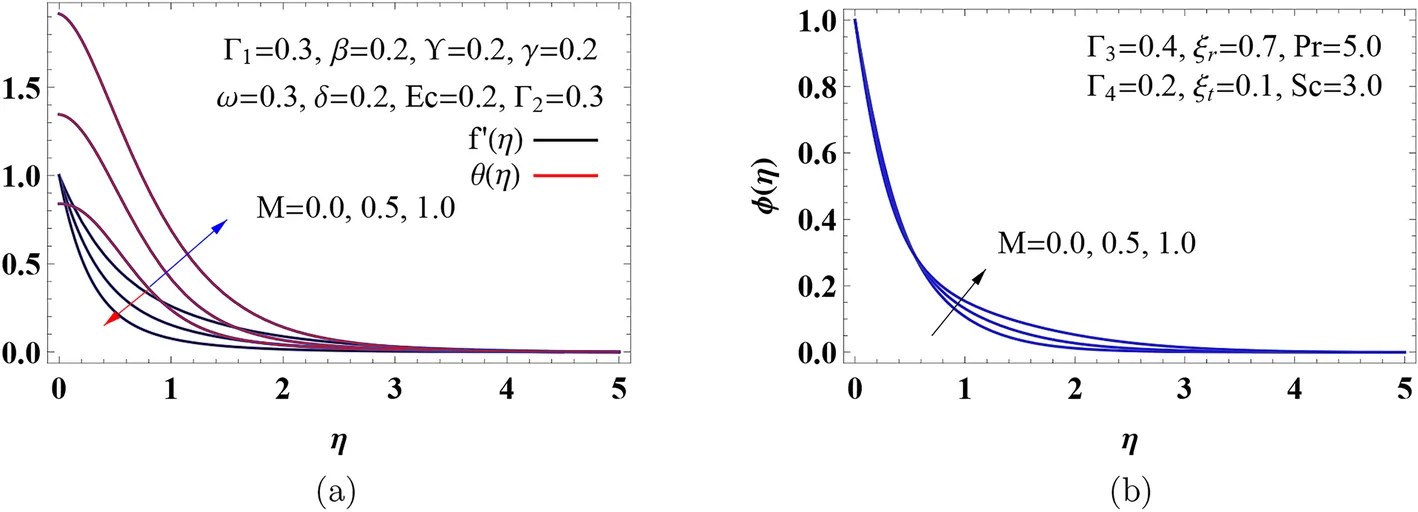
**a** Variation of *M* on *θ*(*η*) and ${f}^{{\prime} }(\eta )$. **b** Variation of *M* on ϕ(*η*)

**Fig. 7:**
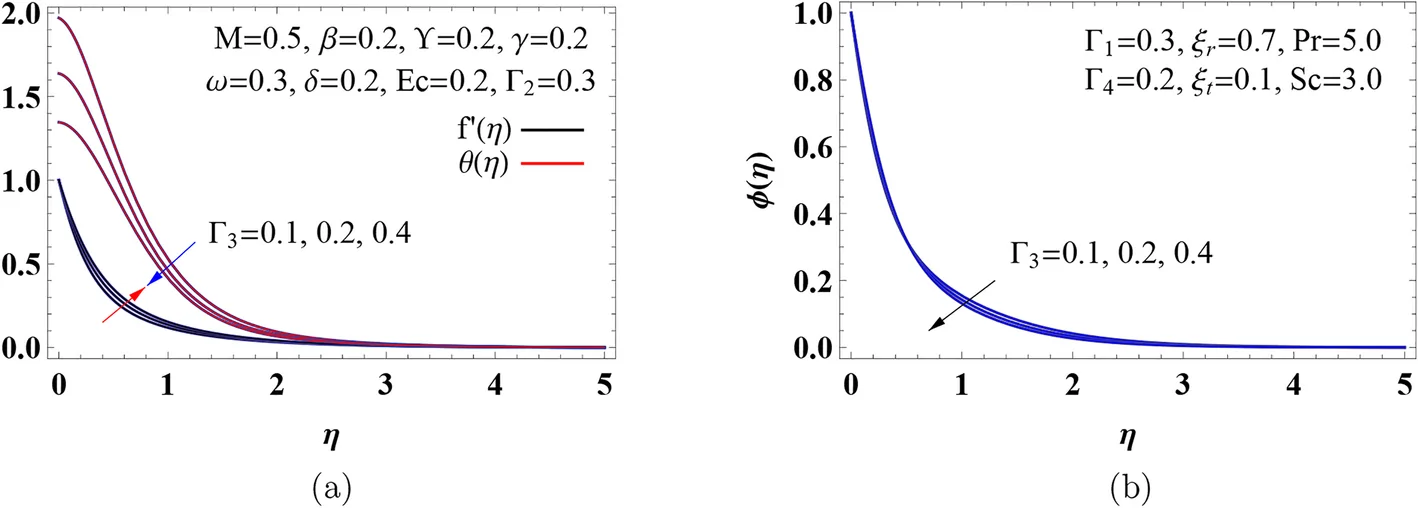
**a** Variation of Γ_3_ on *θ*(*η*) and ${f}^{{\prime} }(\eta )$. **b** Variation of Γ_3_ on ϕ(*η*)

**Fig. 8:**
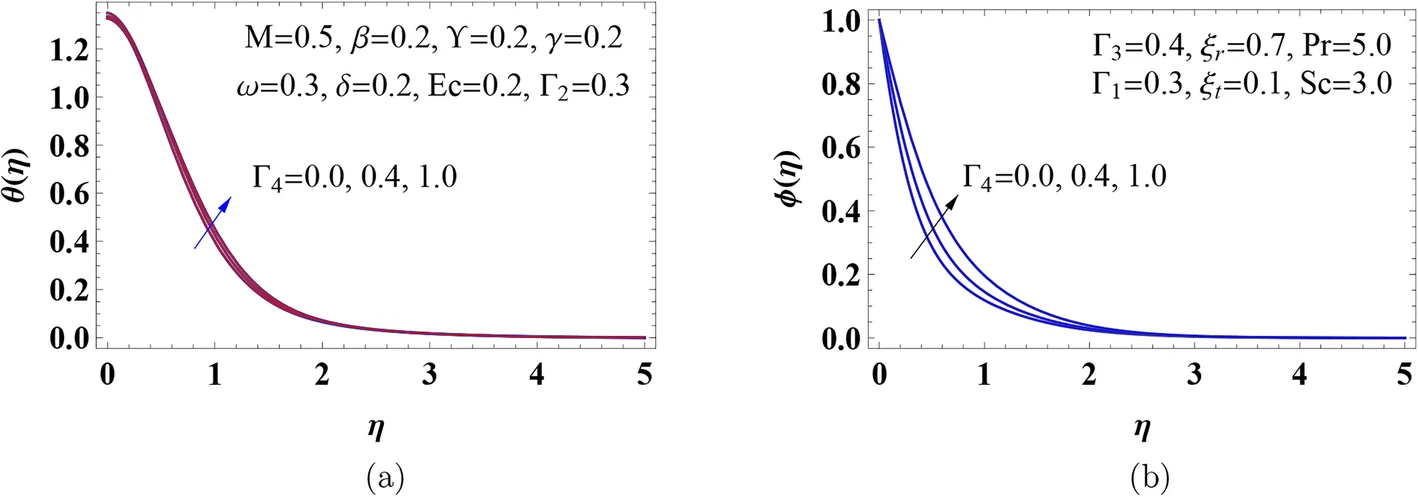
**a** Variation of Γ_4_ on *θ*(*η*). **b** Variation of Γ_4_ on ϕ(*η*)

**Fig. 9:**
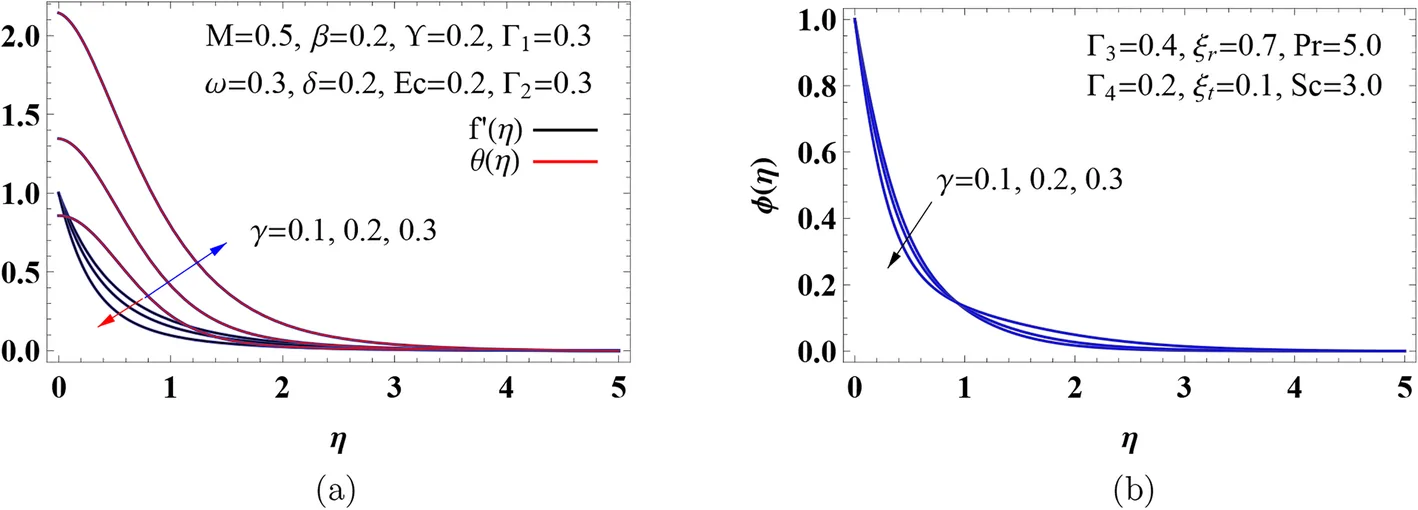
**a** Variation of *γ* on *θ*(*η*) and ${f}^{{\prime} }(\eta )$. **b** Variation of *γ* on ϕ(*η*)

**Fig. 10:**
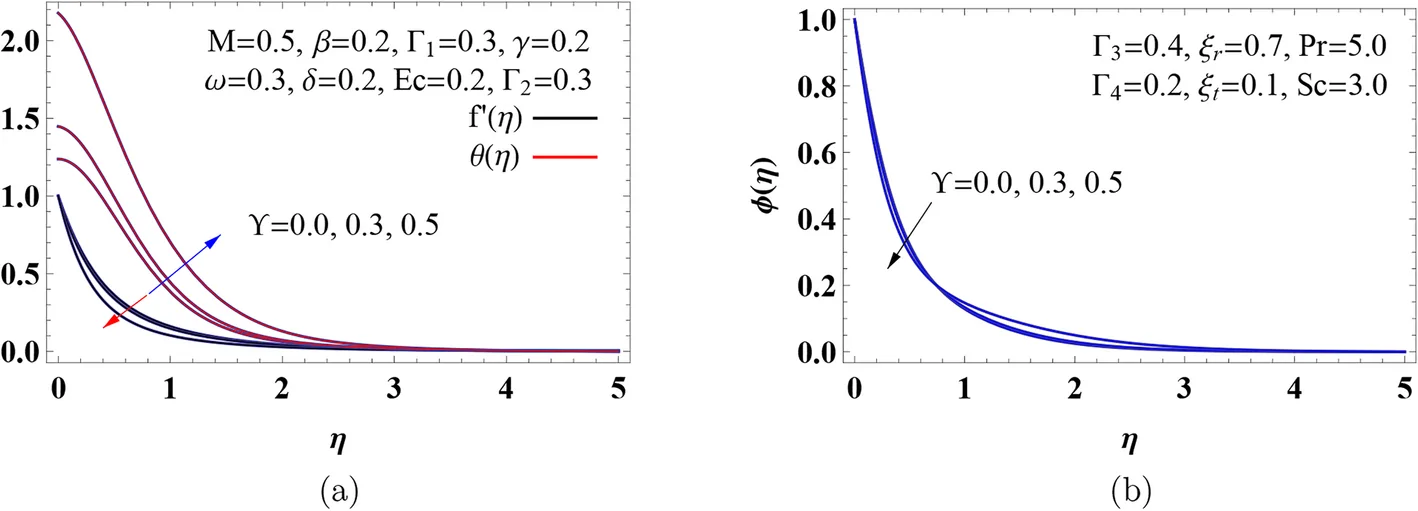
**a** Variation of ϒ on *θ*(*η*) and ${f}^{{\prime} }(\eta )$. **b** Variation of ϒ on ϕ(*η*)

**Fig. 11:**
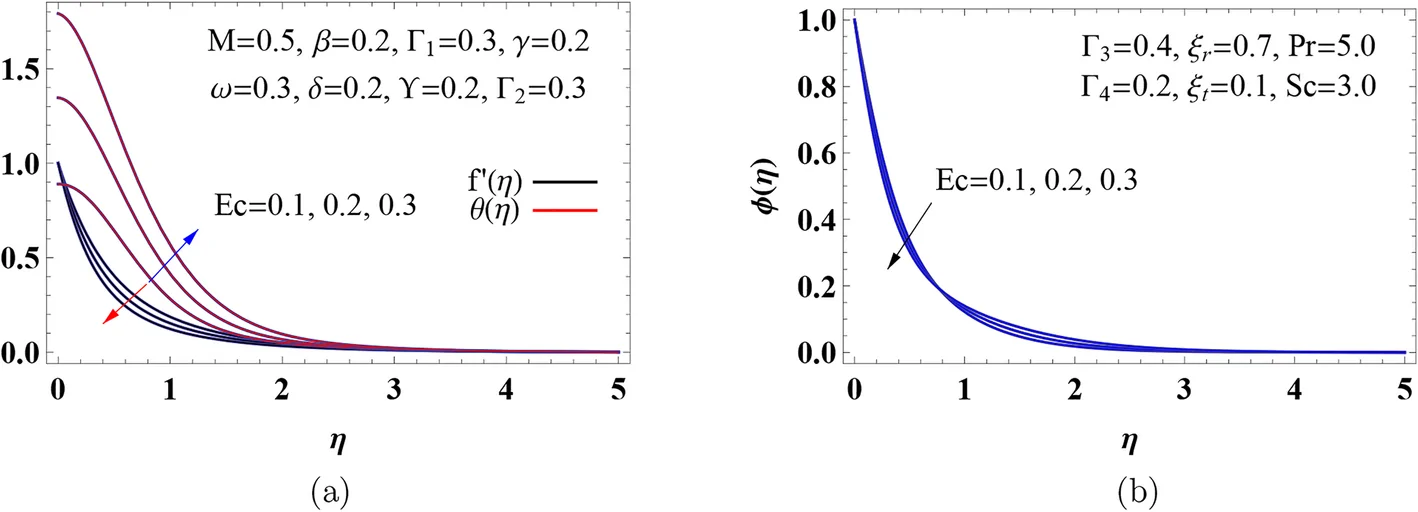
**a** Variation of *Ec* on *θ*(*η*) and ${f}^{{\prime} }(\eta )$. **b** Variation of *Ec* on ϕ(*η*)

The system of Eqs. (13)–(17) is highly nonlinear and does not admit closed-form analytical solutions. For this reason, we employed a numerical shooting method to obtain the solutions. The computed results are presented in the form of plots (Figs. 2–11), illustrating the velocity ${f}^{{\prime} }$, the temperature *θ*, and concentration ϕ(*η*) distributions for various parameter values. These graphical results demonstrate the sensitivity of the solutions to the governing parameters and provide a clear physical interpretation of the model. Therefore, the numerical computations were performed in MATHEMATICA via the shooting technique. This method addresses the boundary value problem by transforming it into an initial value problem and iteratively calibrating the initial guesses to achieve convergence with the required boundary conditions. To facilitate a numerical solution, the previous higher-order differential equations are recast as a system of first-order initial value problems. This is achieved by defining new variables, which decomposes the complex system (13)–(17) into a simpler structure. Proceeding from this framework, we adopt the following assumption:

$$\left\{\begin{array}{lll}f={Z}_{1},&{f}^{{\prime} }={Z}_{2},&{f}^{{\prime\prime} }={Z}_{3},\\ \theta ={Z}_{4},&{\theta }^{{\prime} }={Z}_{5},&\\ \phi ={Z}_{6},&{\phi }^{{\prime} }={Z}_{7}.&\end{array}\right.$$

Consequently, the preceding transformations results in the following system:

20 $$\dfrac{d\,{Z}_{1}}{d\eta }-{Z}_{2}=0,\qquad \quad {Z}_{1}(0)=\omega \,{e}^{-{{\rm{\Gamma }}}_{1}{\epsilon }_{1}},$$

21 $$\dfrac{d\,{Z}_{2}}{d\eta }-{Z}_{3}=0,\qquad \quad {Z}_{2}(0)=1,$$

22 $$\dfrac{d\,{Z}_{4}}{d\eta }-{Z}_{5}=0,\qquad \quad {Z}_{4}(0)={\epsilon }_{1},$$

23 $$\dfrac{d\,{Z}_{6}}{d\eta }-{Z}_{7}=0,\qquad \quad {Z}_{6}(0)=1,$$

24 $$\begin{array}{rcl}{Z}_{3}^{{\prime} }&=&\left(\dfrac{({{\rm{\Gamma }}}_{1}+{{\rm{\Gamma }}}_{2})\,{e}^{-({{\rm{\Gamma }}}_{1}+{{\rm{\Gamma }}}_{2}){Z}_{4}}\,{Z}_{3}{Z}_{5}-\beta \,\left[\left(2\,{Z}_{2}{Z}_{1}+{{\rm{\Gamma }}}_{1}\,{Z}_{1}^{2}\,{Z}_{5}\right){Z}_{3}^{2}\right]+{Z}_{2}^{2}-{Z}_{1}{Z}_{3}}{{e}^{-({{\rm{\Gamma }}}_{1}+{{\rm{\Gamma }}}_{2}){Z}_{4}}-\beta \,{Z}_{1}^{2}}\right)\\ &&+\left(\dfrac{\delta \,{Z}_{2}\,{e}^{-({{\rm{\Gamma }}}_{1}-{{\rm{\Gamma }}}_{2}){Z}_{4}}+M\,{Z}_{2}\,{e}^{{{\rm{\Gamma }}}_{1}{Z}_{4}}}{{e}^{-({{\rm{\Gamma }}}_{1}+{{\rm{\Gamma }}}_{2}){Z}_{4}}-\beta \,{Z}_{1}^{2}}\right),\qquad {Z}_{3}(0)={\epsilon }_{2}\end{array}$$

25 $$\begin{array}{rcl}{Z}_{5}^{{\prime} }&=&\dfrac{\left[\left({{\rm{\Gamma }}}_{1}-{{\rm{\Gamma }}}_{3}+{{\rm{\Gamma }}}_{1}\,{{\rm{\Gamma }}}_{3}\,{Z}_{4}\right){Z}_{5}^{2}\right]-\gamma \,\Pr {e}^{2{{\rm{\Gamma }}}_{1}{Z}_{4}-\eta }-M\,Ec\,\Pr {e}^{2{{\rm{\Gamma }}}_{1}{Z}_{4}}\,{Z}_{2}^{2}-\Pr {Z}_{1}\,{Z}_{5}\,{e}^{{{\rm{\Gamma }}}_{1}{Z}_{4}}}{1+{{\rm{\Gamma }}}_{3}\,{Z}_{4}}\\ &&-\dfrac{Ec\,\Pr {e}^{{{\rm{\Gamma }}}_{1}{Z}_{4}}{Z}_{3}}{1+{{\rm{\Gamma }}}_{3}\,{Z}_{4}}\left[{e}^{-({{\rm{\Gamma }}}_{1}+{{\rm{\Gamma }}}_{2}){Z}_{4}}\,{Z}_{3}+\beta \,{Z}_{1}\,\left(2\,{Z}_{2}^{2}-{Z}_{1}{Z}_{3}\right)\right]\\ &&-\dfrac{\Pr {e}^{-{{\rm{\Gamma }}}_{1}{Z}_{4}}}{1+{{\rm{\Gamma }}}_{3}\,{Z}_{4}}\left[{\xi }_{t}\,{Z}_{5}^{2}+{\xi }_{r}\,{Z}_{5}\,{Z}_{7}\right],\qquad \qquad {Z}_{5}(0)={\rm{\Upsilon }}\,{e}^{{{\rm{\Gamma }}}_{1}{\epsilon }_{1}}\,\left(\dfrac{1-{\epsilon }_{1}}{1+{{\rm{\Gamma }}}_{3}\,{\epsilon }_{1}}\right)\end{array}$$

26 $$\begin{array}{lll}{Z}_{7}^{{\prime} }&=&\dfrac{\left[{{\rm{\Gamma }}}_{1}\left(1+{{\rm{\Gamma }}}_{4}\,{Z}_{7}\right){Z}_{5}\,{Z}_{7}-{{\rm{\Gamma }}}_{4}\,{Z}_{7}^{2}\right]-Sc\,{e}^{2{{\rm{\Gamma }}}_{1}{Z}_{4}}{Z}_{1}\,{Z}_{7}-\left(\dfrac{{\xi }_{t}}{{\xi }_{r}}\right)\left({Z}_{5}^{{\prime} }-{{\rm{\Gamma }}}_{1}\,{Z}_{5}^{2}\right)}{1+{{\rm{\Gamma }}}_{4}\,{Z}_{7}},\\ {Z}_{7}(0)&=&{\epsilon }_{3}.\end{array}$$

A key difficulty in this technique is the appropriate assignment of the values to unknown parameters ϵ_1_, ϵ_2_, and ϵ_3_. The numerical solution’s reliability hinges on this choice, with suboptimal values potentially causing either convergence issues or excessive computational demands. An optimal balance between accuracy and computational efficiency is achieved by setting a tolerance of 10^−7^ and a step size of 0.0001. A solution is deemed convergent when the results satisfy the boundary conditions; otherwise, Newton’s method iteratively refines the initial estimates until convergence is attained. To further verify the numerical stability, computations were repeated using different step sizes and truncated boundary-layer thicknesses. The obtained results remained unchanged up to the reported decimal places, confirming that the numerical solution is insensitive to these computational settings.

## Validation of method accuracy

This section validates the accuracy of the numerical shooting method by comparing the present results with previously published benchmark solutions under different limiting cases. Specifically, the skin-friction coefficient exhibits excellent agreement with the results of Megahed [29] and Abel et al. [34], confirming the reliability of the proposed numerical approach. Furthermore, Tables 1 and 2 include the corresponding relative errors, which are consistently of the order of 10^−5^–10^−4^%, demonstrating excellent quantitative agreement. These negligible deviations confirm the computational robustness, numerical stability, and high accuracy of the proposed numerical scheme, supporting its suitability for modeling complex coupled transport phenomena in non-Newtonian nanofluids.

**Table 1:** Comparison of skin friction coefficient with special values of *β* and *M* when Γ_1_ = Γ_2_ = 0 and *ω* = 0.5

*β*	*M*	Megahed [29]	Present work	Relative error (%)
0.0	1.5	1.3956438	1.39564379021	7.01 × 10^−7^
0.2	1.5	1.4942535	1.49425347707	1.53 × 10^−6^
0.5	1.5	1.6465991	1.64659899084	6.63 × 10^−6^
0.5	1.0	0.9673285	0.96732847799	2.28 × 10^−6^
0.5	1.5	1.6465991	1.64659899084	6.63 × 10^−6^
0.5	2.0	2.1869332	2.18693319805	8.92 × 10^−8^

**Table 2:** Comparison of skin friction coefficient with special values of *β* when *δ* = *ω* = Γ_1_ = Γ_2_ = 0.0 and *M* = 0.2

*β*	Abel et al. [34]	Present work	Relative error (%)
0.0	1.095445	1.095444659	3.11 × 10^−5^
0.2	1.188270	1.188268808	1.00 × 10^−4^
0.4	1.275878	1.275877597	3.16 × 10^−5^
0.4	1.358733	1.358729907	2.28 × 10^−4^

## Findings and interpretation

This section decodes the intricate interplay of physical phenomena within a Maxwell nanofluid flowing over a stretching surface. We integrate and analyze five key coupled mechanisms: convective boundary heating, internal energy generation, Ohmic dissipation, temperature-sensitive properties (viscosity, density, diffusivity), and viscous thermal effects, revealing how these forces collectively shape flow dynamics, thermal distribution, and mass transfer in ways previously unexamined. This study harmonizes a suite of twelve dimensionless parameters, each representing a distinct physical force-to fully characterize nanofluid behavior. From the fluid’s elastic memory (Maxwell parameter *β*) and magnetic influence *M* to molecular diffusion (Schmidt *Sc*, Prandtl Pr), thermophoretic *ξ*_*t*_ and Brownian nanoparticle *ξ*_*r*_ dynamics, convective energy exchange ϒ, the heat source/sink parameter *γ*, viscous heating (Eckert *Ec*), and boundary suction *ω*, each parameter contributes a unique voice to the collective description of flow, thermal, and concentration fields. Now and based on the following Fig. 2, the curves depict the variations of velocity ${f}^{{\prime} }(\eta )$, temperature *θ*(*η*), and concentration ϕ(*η*) profiles for different values of the porous parameter *δ*. The results indicate that an increase in the porous parameter *δ* leads to a reduction in the velocity profile. This occurs because higher *δ* introduces more resistance within the porous medium, which opposes the fluid flow and thus decelerates it. Conversely, raising *δ* elevates both *θ*(*η*) and ϕ(*η*) distributions, though the latter rises only marginally. Physically, the temperature growth phenomenon stems from energy dissipation that induced by porous resistance, which converts kinetic energy into heat. The slight concentration increase is linked to slower fluid motion, limiting dispersion and enabling minor solute buildup. Essentially, the porosity parameter reduces medium permeability, where greater porosity imposes more resistance, slowing the flow while retaining heat and solute through weaker convective transport.

Figure 3 shows how the temperature-dependent density parameter Γ_1_ affects the velocity ${f}^{{\prime} }(\eta )$, temperature *θ*(*η*), and concentration ϕ(*η*) of the Maxwell nanofluid. As this parameter Γ_1_ increases (from 0.0 to 0.4), the fluid’s velocity slows down throughout the boundary layer. This happens because Γ_1_ represents thermal buoyancy, and a greater value of it indicates a stronger buoyant force that works against the fluid’s motion. This opposing force acts like a resistance, causing the fluid to decelerate. On the other hand, *θ*(*η*) profile shows a notable rise, especially near the sheet surface, due to the stronger buoyancy-driven effects that increase the mechanism of energy transfer. This results in more thermal energy being generated and a slower cooling rate. As for the concentration profile, ϕ(*η*) leads to a decrease, especially near the sheet’s surface, which can be explained by the opposing buoyancy force that impedes the mass diffusion mechanism near the surface. However, farther from the sheet surface, this impact is somewhat reversed at *η* > 1.5, as the altered flow structure and the dominance of convective transport begin to outweigh the diffusive effects. Physically, increasing the density parameter strengthens the wall shear, leading to a higher skin-friction coefficient. The enhanced wall resistance suppresses the fluid motion while promoting thermal energy accumulation and reducing nanoparticle diffusion within the boundary layer.

Based on the following Fig. 4, the curves illustrate the effect of the variable viscosity parameter Γ_2_ on the ${f}^{{\prime} }(\eta )$, *θ*(*η*) and ϕ(*η*) profiles. The results show that increasing Γ_2_ (from 0.0 to 0.2 and 0.5) results in a reduction in the ${f}^{{\prime} }(\eta )$ profile throughout the entire boundary layer. This is because a higher Γ_2_ corresponds to an enhance in nanofluid viscosity. Then the increased viscosity intensifies the internal resistive forces within the layers of the fluid, which oppose its motion and consequently slow down the flow. Conversely, both the temperature and concentration profiles are enhanced with increasing Γ_2_. Nevertheless, the influence on the thermal profile *θ*(*η*) is more dominant than on the concentration profile ϕ(*η*) because the direct impact of Γ_2_ on the thermal field. Further, the pronounced elevation in temperature arises mainly from the effect of viscous dissipation phenomenon. As viscosity grows, a greater portion of mechanical energy is transformed to thermal heat through frictional forces, which is reflected in the energy equation by the Eckert number (*Ec* = 0.2). Consequently, this mechanism significantly intensifies the thermal boundary layer. Physically, variations in viscosity modify the fluid’s resistance to motion. Higher viscosity increases heat generation through dissipation, which accounts for the pronounced thermal response. In contrast, its influence on concentration is subdued, arising indirectly from changes in velocity gradients.

The analysis of Fig. 5 clearly demonstrates the significant role of the suction parameter *ω* in modulating the boundary layer characteristics. An increase in *ω* is observed to enhance the fluid velocity ${f}^{{\prime} }(\eta )$ adjacent to the surface. This acceleration is attributed to the thickening of the boundary layer, which improves momentum transfer. In contrast, the temperature profile *θ*(*η*) exhibits a substantial reduction with stronger suction. This cooling effect arises as the removal of fluid particles directly extracts thermal energy from the boundary layer. Further, the influence on concentration ϕ(*η*), while present, is more moderate. The thinning of the boundary layer under suction restricts mass diffusion, though this effect is less pronounced than the thermal response. Physically, suction serves as a stabilizing mechanism that concurrently accelerates the flow, enhances cooling, and moderately reduces solute concentration near the wall.

The results from Fig. 6 distinctly illustrate the impact of the magnetic field parameter *M* on the flow velocity ${f}^{{\prime} }(\eta )$ and transport characteristics in terms of *θ*(*η*) and ϕ(*η*). An increase in *M* diminishes the velocity profile ${f}^{{\prime} }(\eta )$ throughout the boundary layer. This behavior results from the Lorentz force, which counteracts the fluid motion and functions as a resistive drag, thereby slowing down the flow. Conversely, the temperature profile *θ*(*η*) experiences a significant enhancement. The heightened temperature arises from increased Joule heating and viscous dissipation associated with the magnetic field, which converts kinetic energy into thermal energy. The concentration profile ϕ(*η*) shows a slight rise with increasing *M*, though this behavior is much weaker than the thermal response and becomes noticeable mainly at points farther from the sheet surface *η* > 0.6. Close to the wall *η* < 0.6, its influence is very minimal because the competing effects of molecular diffusion and flow retardation balance each other. In regions away from the sheet surface, however, the slower fluid motion permits greater accumulation of nanoparticles. Physically, greater value of *M* suppresses momentum transport and enhances thermal energy generation, whereas its impact on mass transfer remains relatively minor and varies with spatial location.

Figure 7 clearly demonstrates that the variable thermal conductivity parameter Γ_3_ plays a crucial role in shaping the flow characteristics of the Maxwell nanofluid. An increase in Γ_3_ results in a clear decrease in the temperature distribution *θ*(*η*) throughout the boundary layer, with the most pronounced cooling observed close to the plate. This effect arises because larger values of Γ_3_ enhance the fluid’s thermal conductivity, allowing heat to dissipate more efficiently from the surface and thereby strengthening thermal diffusion and cooling. Similarly, the concentration profile ϕ(*η*) decreases with rising the same parameter Γ_3_, although this effect is more subtle and is primarily evident further away from the sheet surface. The reduction in concentration is a secondary consequence of the modified thermal field and altered flow structure. Conversely, the velocity profile exhibits an increase with higher Γ_3_. This acceleration is physically attributed to the changes in fluid properties induced by the enhanced thermal conductivity, which reduce the overall viscous resistance and facilitate fluid motion. Essentially, the parameter Γ_3_ strengthens thermal diffusion, leading to efficient cooling, a mild suppression of concentration, and a supportive effect on the flow velocity.

The results from Fig. 8 demonstrate the significant impact of the variable mass diffusivity parameter Γ_4_ on the concentration ϕ(*η*) and thermal *θ*(*η*) fields. With higher values of Γ_4_, the concentration profile ϕ(*η*) shows a marked rise throughout the boundary layer, most notably in the region close to the sheet. This behavior is attributed to the fact that larger Γ_4_ enhances the fluid’s mass diffusion capacity, thereby promoting more effective transport and accumulation of solute particles near the wall. Further, the temperature profile *θ*(*η*) also exhibits a slight increase in response to elevated, although this thermal response is considerably weaker. The mild rise in *θ*(*η*) is an indirect effect, primarily resulting from the coupling between the concentration and thermal fields through cross-diffusion mechanisms. Physically, the parameter enhances mass transfer efficiency, leading to substantial solute build-up near the boundary, while its secondary influence on temperature stems from interrelated energy and mass transport processes.

Figure 9 illustrates the influence of the heat generation parameter *γ* on *θ*(*η*), ϕ(*η*) and ${f}^{{\prime} }(\eta )$ profiles. As *γ* increases, the velocity within the boundary layer decreases notably, since part of the generated energy is transformed into internal thermal energy rather than kinetic energy, which stabilizes the flow and diminishes fluid motion. Conversely, the temperature profile exhibits a substantial and pronounced increase, particularly near the sheet surface. This sharp rise is a direct result of the internal heat generation, which introduces additional thermal energy into the system, enhancing the thermal boundary layer. The concentration response is more complex, because close to the plate, concentration is reduced due to dominant thermal influences altering local diffusion rates, while away the sheet surface, a slight increase appears, likely caused by convective adjustments in both *θ*(*η*) and ${f}^{{\prime} }(\eta )$ fields that permit solute buildup. Physically, heat generation mechanism serves primarily as a thermal energy source that strengthens temperature growth and flow stability, while exerting only an indirect and relatively weak influence on mass transfer.

Figure 10 highlights the strong influence of the thermal convection parameter ϒ on the behavior of the boundary layer profiles in terms of *θ*(*η*), ϕ(*η*) and ${f}^{{\prime} }(\eta )$. Raising ϒ produces a clear decline in fluid velocity ${f}^{{\prime} }(\eta )$ throughout the boundary layer. This slowdown arises from the intensified thermal buoyancy force at higher ${f}^{{\prime} }(\eta )$, which counteracts the main flow direction and introduces a resistive effect that diminishes the fluid motion. In contrast, the temperature distribution *θ*(*η*) shows a marked enhancement, most evident near the sheet surface. This rise in heating results from the stronger convective energy transfer, especially at higher ϒ, which enhances thermal diffusion and promotes greater energy retention within the boundary layer. Further, the concentration profile ϕ(*η*) exhibits a dual response: in the vicinity of the wall surface, it decreases because the modified thermal and velocity fields strongly affect mass diffusion. However, farther from the wall surface, a modest rise in concentration appears, which can be linked to the altered flow structure that weakens convective mass transfer and permits limited solute buildup. Physically, the parameter ϒ intensifies thermal energy via buoyancy-induced convection, which not only slows down the fluid motion but also exerts a spatially dependent effect on mass distribution relative to the boundary.

An examination of the provided data in the following Fig. 11 shows a clear relationship between the Eckert number *Ec* and the behavior of ϕ(*η*), *θ*(*η*) and ${f}^{{\prime} }(\eta )$. As *Ec* increases, there’s a notable decrease in the velocity ${f}^{{\prime} }(\eta )$ of the Maxwell nanofluid. This happens because higher *Ec* signify more viscous heating, meaning more of the fluid’s kinetic energy is converted into heat through friction, which slows the flow down. In contrast, the temperature of the fluid *θ*(*η*) rises dramatically, especially close to the wall surface. This is a direct result of the viscous dissipation phenomenon, where the work done against fluid friction turns into thermal energy, causing the temperature to spike. The concentration behavior that described through ϕ(*η*) profile is relatively intricate: close to the wall surface it declines under the influence of strong thermal effects, whereas at greater distances *η* > 0.8 it shows a modest rise. Physically, this pattern can be explained by the altered flow structure, which weakens convective mass transport in the outer boundary layer, allowing limited solute accumulation.

Now and based on the comprehensive parametric study presented in the following Table 3, the influence of various physical parameters on the skin friction coefficient $C{f}_{x}\sqrt{R{e}_{x}}$, Nusselt number $N{u}_{x}R{e}_{x}^{\frac{-1}{2}}$ and Sherwood number $S{h}_{x}R{e}_{x}^{\frac{-1}{2}}$ is clearly demonstrated. These engineering quantities provide direct measures of surface drag, heat transfer efficiency, and mass transport performance, making them key indicators for the design and optimization of practical thermal systems. The porous parameter *δ* enhances skin friction due to increased flow resistance within the porous medium, while also increasing both heat and mass transfer rates as a result of reduced boundary layer thickness. The temperature-dependent density parameter Γ_1_ significantly amplifies skin friction and both the Sherwood number and the Nusselt numbers, owing to strengthened buoyancy effects that improve convective transport, though its impact on mass transfer is comparatively moderate. An increase in the variable viscosity parameter Γ_2_ raises both the Nusselt and the skin friction due to heightened fluid resistance, but reduces the Sherwood numbers as viscous effects suppress solutal diffusion. Likewise, the magnetic parameter *M* elevates skin friction through Lorentz force-induced resistance, and increases thermal and mass diffusion slightly due to Joule heating and modified flow structure. A reduction in variable thermal conductivity Γ_3_ increases the Nusselt number by enhancing heat conduction mechanism, while increasing skin friction and noticeably enhancing mass transfer. Further, he suction factor *ω* markedly decreases skin friction and heat transfer by thinning the boundary layer thickness, while its effect on mass transfer is pronounced by increase behavior. Higher variable mass diffusivity Γ_4_ reduces the Sherwood number by promoting mass diffusion away from the surface, with minimal impact on skin friction and heat transfer by increasing behavior. The heat generation parameter *γ* strongly enhances the Nusselt number by introducing internal energy, but increases skin friction and slightly enhances mass transfer. Additionally, the convective boundary condition parameter ϒ significantly boosts the Nusselt number by strengthening thermal exchange at the wall, while also increasing skin friction and mass transfer. The enhanced heat transfer characteristics associated with higher Nusselt numbers are particularly beneficial for improving the thermal efficiency of heat exchangers and other thermal management devices. Finally, the Eckert number *Ec* increases both skin friction and the Nusselt number due to viscous dissipation converting kinetic energy into heat, well as it slightly enhances mass transfer as flow modification affects solute distribution. Overall, the obtained trends provide quantitative guidance for balancing heat transfer enhancement, mass transport, and surface drag, thereby supporting the optimization of polymer processing, heat exchangers, and nanofluid-based thermal management systems.

**Table 3:** Values of $C{f}_{x}\sqrt{R{e}_{x}}$, $S{h}_{x}R{e}_{x}^{\frac{-1}{2}}$ and $N{u}_{x}R{e}_{x}^{\frac{-1}{2}}$ for distinct values of the governing factors with *Sc* = 3.0, *ξ*_*r*_ = 0.7, *ξ*_*t*_ = 0.1, *β* = 0.2 and *Pr* = 5.0

*δ*	Γ_1_	Γ_2_	*ω*	*M*	Γ_3_	Γ_4_	*γ*	ϒ	*Ec*	$C{f}_{x}\sqrt{R{e}_{x}}$	$N{u}_{x}R{e}_{x}^{\frac{-1}{2}}$	$S{h}_{x}R{e}_{x}^{\frac{-1}{2}}$
0.0	0.3	0.3	0.3	0.5	0.4	0.2	0.2	0.2	0.2	2.47222	0.1021271	2.15882
0.2	0.3	0.3	0.3	0.5	0.4	0.2	0.2	0.2	0.2	2.67653	0.1205680	2.18368
0.5	0.3	0.3	0.3	0.5	0.4	0.2	0.2	0.2	0.2	2.98583	0.1486852	2.22437
0.2	0.0	0.3	0.3	0.5	0.4	0.2	0.2	0.2	0.2	1.89024	0.0495036	1.43954
0.2	0.2	0.3	0.3	0.5	0.4	0.2	0.2	0.2	0.2	2.67629	0.1205520	2.18362
0.2	0.4	0.3	0.3	0.5	0.4	0.2	0.2	0.2	0.2	3.49812	0.2375121	2.86679
0.2	0.3	0.0	0.3	0.5	0.4	0.2	0.2	0.2	0.2	1.98199	0.1062271	2.24974
0.2	0.3	0.2	0.3	0.5	0.4	0.2	0.2	0.2	0.2	2.41153	0.1145690	2.20558
0.2	0.3	0.5	0.3	0.5	0.4	0.2	0.2	0.2	0.2	3.39264	0.1404581	2.14501
0.2	0.3	0.3	0.2	0.5	0.4	0.2	0.2	0.2	0.2	3.06990	0.2194341	2.13257
0.2	0.3	0.3	0.3	0.5	0.4	0.2	0.2	0.2	0.2	2.67549	0.1203830	2.18284
0.2	0.3	0.3	0.4	0.5	0.4	0.2	0.2	0.2	0.2	2.48541	0.0629966	2.30525
0.2	0.3	0.3	0.3	0.0	0.4	0.2	0.2	0.2	0.2	1.83719	0.0173816	1.99478
0.2	0.3	0.3	0.3	0.5	0.4	0.2	0.2	0.2	0.2	2.67549	0.1203830	2.18284
0.2	0.3	0.3	0.3	1.0	0.4	0.2	0.2	0.2	0.2	3.39005	0.2008973	2.35072
0.2	0.3	0.3	0.3	0.5	0.1	0.2	0.2	0.2	0.2	4.68505	0.3927601	2.71029
0.2	0.3	0.3	0.3	0.5	0.2	0.2	0.2	0.2	0.2	3.15312	0.2714901	2.38759
0.2	0.3	0.3	0.3	0.5	0.4	0.2	0.2	0.2	0.2	2.67549	0.1203830	2.18284
0.2	0.3	0.3	0.3	0.5	0.4	0.0	0.2	0.2	0.2	2.66349	0.1174952	2.55954
0.2	0.3	0.3	0.3	0.5	0.4	0.4	0.2	0.2	0.2	2.68081	0.1217820	1.90646
0.2	0.3	0.3	0.3	0.5	0.4	1.0	0.2	0.2	0.2	2.69592	0.1374393	1.37884
0.2	0.3	0.3	0.3	0.5	0.4	0.2	0.1	0.2	0.2	1.71399	0.0076572	1.87963
0.2	0.3	0.3	0.3	0.5	0.4	0.2	0.2	0.2	0.2	2.67549	0.1203830	2.18284
0.2	0.3	0.3	0.3	0.5	0.4	0.2	0.3	0.2	0.2	2.83403	0.2342780	2.57707
0.2	0.3	0.3	0.3	0.5	0.4	0.2	0.2	0.0	0.2	1.99156	0.0000001	2.06776
0.2	0.3	0.3	0.3	0.5	0.4	0.2	0.2	0.3	0.2	2.17018	0.1311630	2.15887
0.2	0.3	0.3	0.3	0.5	0.4	0.2	0.2	0.5	0.2	2.90147	0.6042751	2.52248
0.2	0.3	0.3	0.3	0.5	0.4	0.2	0.2	0.2	0.1	1.73084	0.0113701	1.90183
0.2	0.3	0.3	0.3	0.5	0.4	0.2	0.2	0.2	0.2	2.67549	0.1203830	2.18284
0.2	0.3	0.3	0.3	0.5	0.4	0.2	0.2	0.2	0.3	2.88351	0.1579651	2.34864

## Concluding remarks

This study pioneers a holistic model for Maxwell nanofluid flow, integrating convection, Ohmic heating, variable properties, and dissipation into a unified framework. The numerical approach proved robust and accurate. A limitation of the present study is that it is restricted to steady two-dimensional flow under boundary-layer assumptions, without considering transient or three-dimensional effects. Key findings reveal that:

- The temperature-dependent density parameter intensifies the thermal field while diminishing nanofluid velocity and concentration, and simultaneously enhances heat/mass transfer rates and skin friction.
- Heat generation and convection parameters amplify the temperature distribution but suppress fluid motion, leading to reduced velocity.
- The suction parameter acts as a kinetic amplifier, accelerating fluid motion while simultaneously suppressing thermal and concentration levels. This dynamic leads to a notable reduction in skin friction and heat transfer efficiency. Conversely, mass transfer rates experience a significant boost.
- The thermal conductivity parameter acts as a hidden accelerator for fluid motion while simultaneously dampening thermal and solutal distributions. This dual behavior effectively reduces skin friction drag and attenuates both heat and mass transfer rates.
- The Eckert number serves as a transformative agent: it suppresses flow momentum while significantly elevating nanofluid temperature. This thermal amplification, driven by viscous dissipation, subsequently increases skin friction resistance and amplifies both heat and mass transfer rates.
- The observed increase in thermal distribution with the porous, density, viscosity, magnetic field, and heat generation parameters supports the elevated operating temperatures required during polymer processing and contributes to improved thermal management in heat exchangers and nanofluid-based cooling systems.
- In future studies, the present model may be extended to transient three-dimensional Maxwell nanofluid flows with additional effects, such as nonlinear thermal radiation, velocity slip, and hybrid nanofluids. Physics-informed deep learning may also be explored to solve more complex coupled transport problems.

## Data Availability

All data generated or analysed during this study are included in this published article.

## Nomenclature

*a*: Constant (s^−1^)
*T*: Temperature (K)
*B*_0_: Magnetic field strength (T)
*E**c*: Eckert number
$${Q}^{{\prime\prime} {\prime} }$$: Heat generation (W m^−3^)
*C*_*w*_: Coefficient of nanoparticle along the sheet (mol L^−1^)
*D*_*B*_: Coefficient of Brownian diffusion (m^2^ s^−1^)
Pr: Prandtl number
*c*_*p*_: Specific heat at constant pressure (J kg^−1^ K^−1^)
*T*_*f*_: Temperature of heating fluid (K)
*D*_*T*_: Coefficient of thermophoretic diffusion (m^2^ s^−1^)
*C*_*∞*_: Coefficient of Nanoparticle concentration at infinity (*m**o**l**L*^−1^)
*M*: Magnetic field parameter
*f*: Dimensionless stream function
*T*_*∞*_: Temperature of fluid at infinity (K)
*C*: Density of nanoparticle (mol L^−1^)
*u*, *v*: Velocity components (m s^−1^)
*v*_*w*_: Suction velocity (m s^−1^)
Γ_1_: Density factor
Γ_2_: Viscosity factor
Γ_3_: Thermal conductivity factor
Γ_4_: Diffusion factor
*η*: Dimensionless variable
*θ*: Dimensionless temperature
*ω*: Suction factor
ϒ: Convection parameter
*γ*: Heat generation/absorption factor
ϕ: Dimensionless concentration
*δ*: Porous parameter
*β*: Maxwell parameter
*ξ*_*r*_: Brownian motion parameter
*μ*_0_: Reference nanofluid viscosity (kg m^−1^ s^−1^)
*κ*: Thermal conductivity (W m^−1^ K^−1^)
*ξ*_*t*_: Thermophoresis parameter
*ρ*: Density (kg m^−3^)
*σ*: Electric conductivity (S m^−1^)
*κ*_0_: Reference thermal conductivity (W m^−1^ K^−1^)
*μ*: Nanofluid viscosity (kg m^−1^ s^−1^)
